# Activated Protein C Attenuates Experimental Autoimmune Encephalomyelitis Progression by Enhancing Vascular Integrity and Suppressing Microglial Activation

**DOI:** 10.3389/fnins.2020.00333

**Published:** 2020-04-15

**Authors:** Ravi Kant, Sebok K. Halder, Jose A. Fernández, John H. Griffin, Richard Milner

**Affiliations:** Department of Molecular Medicine, The Scripps Research Institute, La Jolla, CA, United States

**Keywords:** activated protein C, experimental autoimmune encephalomyelitis, microglia, blood-brain barrier, vascular, matrix metalloproteinase-9

## Abstract

**Background:**

Activated protein C (APC), a serine protease with antithrombotic effects, protects in animal models of ischemic stroke by suppressing inflammation and enhancing vascular integrity, angiogenesis, neurogenesis and neuroprotection. A small number of animal studies suggest it might also have therapeutic potential in multiple sclerosis (MS), though results have been mixed. Based on these conflicting data, the goals of this study were to clarify the therapeutic potential of APC in the experimental autoimmune encephalomyelitis (EAE) model of MS and to determine mechanistically how APC mediates this protective effect.

**Methods:**

The protective potential of APC was examined in a chronic progressive model of EAE. Vascular breakdown, tight junction protein expression and vascular expression of fibronectin and α5β1 integrin as well as vascularity and glial activation were analyzed using immunofluorescence (IF) of spinal cord sections taken from mice with established EAE. The direct influence of APC on microglial activation was evaluated *in vitro* by a combination of morphology and MMP-9 expression.

**Results:**

APC attenuated the progression of EAE, and this was strongly associated at the histopathological level with reduced levels of leukocyte infiltration and concomitant demyelination. Further analysis revealed that APC reduced vascular breakdown which was associated with maintained endothelial expression of the tight junction protein zonula occludens-1 (ZO-1). In addition, APC suppressed microglial activation in this EAE model and *in vitro* studies revealed that APC strongly inhibited microglial activation at both the morphological level and by the expression of the pro-inflammatory protease MMP-9.

**Conclusion:**

These findings build on the work of others in demonstrating strong therapeutic potential for APC in the treatment of inflammatory demyelinating disease and suggest that enhancement of vascular integrity and suppression of microglial activation may be important mediators of this protection.

## Introduction

Multiple sclerosis (MS) is an autoimmune disease of the central nervous system (CNS) and is the most common neurological disease in the young to middle age population, affecting more than 400,000 people in the United States (Wingerchuk and Carter, 2014; Doshi and Chataway, 2017). While the precise trigger of MS is unknown, it typically presents with a spectrum of neurological symptoms that include blindness, altered sensation, weakness and eventually paralysis and cognitive defects. At the pathological level, MS is characterized by infiltration of inflammatory lymphocytes directed against myelin antigens into the CNS (brain and spinal cord), which establishes a chronic inflammatory response, resulting in stripping of the myelin sheath from myelinated axons (demyelination) and ultimately, axonal degeneration (ffrench-Constant, 1994; Lassmann, 1998). Because MS is such a common disease that strikes relatively early in life, the last 30 years has witnessed intensive research activity seeking to understand disease pathogenesis. This activity has generated a large number of disease-modifying therapies (DMTs), several of which are effective at suppressing the inflammatory aspect of MS. To date, most of these drugs have been designed to target cells of the immune system, suppressing their activation, proliferation, or egression from peripheral lymph nodes or their transmigration into the CNS. It should be noted that while each of the drugs offer significant benefits in certain sub-populations of MS patients, because of the heterogeneity of the disease, none of the drugs is effective in all patients, and in some types of MS, particularly the chronic progressive form, few therapeutic options are currently available (Wingerchuk and Carter, 2014; Doshi and Chataway, 2017).

Activated protein C (APC) is a homeostatic serine protease that was first defined as an antithrombotic on the basis of inactivation of the clotting factors Va and VIIIa (Mosnier et al., 2007; Rezaie, 2011; Esmon, 2012). Studies in animal models of neurological disease, particularly ischemic stroke, have shown that in addition to its natural antithrombotic actions, APC also triggers a number of protective actions that include attenuation of neuroinflammation and promotion of vascular integrity, angiogenesis, neurogenesis, and neuroprotection (Joyce et al., 2001; Cheng et al., 2003; Guo et al., 2004; Fernandez et al., 2005; Mosnier and Griffin, 2007; Thiyagarajan et al., 2008; Petraglia et al., 2010; Guo et al., 2013; Wang et al., 2013a, b). Sophisticated molecular dissection studies have shown that the antithrombotic and neuroprotective influences reside in separate domains of the APC protein structure and this has led to the generation of different APC variants that lack these specific activities (Mosnier et al., 2004, 2007). Of particular clinical interest is the 3K3A-APC variant which retains neuroprotective actions but lacks antithrombotic activity, thus avoiding the unwanted side-effect of excess bleeding (Mosnier et al., 2007). Encouraged by a large number of studies demonstrating that APC protects in animal models of ischemic stroke, 3K3A-APC is currently being evaluated in an NIH-funded phase 2 clinical trial of ischemic stroke (RHAPSODY).

Interestingly, a small number of studies in the experimental autoimmune encephalomyelitis (EAE) mouse model of MS have suggested that APC might also hold therapeutic potential in the treatment of MS, though to date, results have been conflicting. Specifically, while approaches designed to increase endogenous APC levels protected against EAE (Verbout et al., 2015; Wolter et al., 2016), surprisingly, antibody neutralization of endogenous APC achieved the same outcome (Alabanza et al., 2013). Furthermore, while exogenous APC demonstrated protection in the relapsing-remitting form of EAE (Han et al., 2008), its effect in the chronic progressive form of EAE has yet to be assessed. Based on this incomplete and at times conflicting data, we embarked on the current study with the objective of clarifying whether APC protects against demyelinating disease, as well as seek to define the molecular mechanisms underlying this protection. In particular, in light of the importance of vascular breakdown and remodeling in the pathogenesis of EAE (Gay and Esiri, 1991; Kirk et al., 2003; Roscoe et al., 2009; Seabrook et al., 2010), and our recent finding that APC promotes physiological cerebrovascular remodeling (Burnier et al., 2016), we specifically wanted to examine whether APC protects in EAE via promotion of vascular remodeling. Thus, this study had the following specific goals: (i) evaluate the therapeutic potential of APC in the chronic progressive form of EAE, and (ii) examine how APC influences pathogenic events at the cellular level, paying particular attention to its effects on vascular breakdown and remodeling, leukocyte infiltration, loss of endothelial tight junction proteins, and glial activation.

## Materials and Methods

### Animals

The studies described have been reviewed and approved by The Scripps Research Institute (TSRI) Institutional Animal Care and Use Committee. Wild-type female C57BL6/J mice were obtained from the TSRI rodent breeding colony and maintained under pathogen-free conditions in the closed breeding colony of TSRI.

### Experimental Autoimmune Encephalomyelitis (EAE)

EAE was performed using a protocol and materials provided by Hooke Laboratories (Lawrence, MA). Briefly, 10-week old C57BL/6 female mice were immunized subcutaneously with 200 μl of 1 mg/ml MOG_35__–__55_ peptide emulsified in complete Freud’s adjuvant (CFA) containing 2 mg/ml Mycobacterium tuberculosis in both the base of the tail and upper back. In addition, on days 0 and 1, mice also received an intraperitoneal injection of 200 ng pertussis toxin. In WT mice this protocol leads to robust induction of clinical EAE on days 12–14 following immunization (Crocker et al., 2006; Milner et al., 2007). Animals were monitored daily for clinical signs and scored as follows: 0-no symptoms; 1-flaccid tail; 2-paresis of hind limbs; 3-paralysis of hind limbs; 4-quadriplegia; 5-death. When mice reached a clinical score of 2 (equivalent to hindlimb weakness), they were randomly allocated to one of two different treatment groups: to receive twice-daily intraperitoneal injections of PBS vehicle or recombinant murine 3K3A-APC (0.2 mg/kg re-suspended in PBS pH 7.4 buffer, 100 μl) which was produced and purified as previously described (Fernandez et al., 2005). This treatment was maintained for the duration of the experiment (2 weeks) and mice were clinically evaluated and scored daily. Mice were euthanized at different time-points of EAE, including day 0 (disease-free control) and 25 days post-immunization (peak disease) to obtain tissue for histological studies.

### Immunohistochemistry and Antibodies

Immunohistochemistry was performed as described previously (Milner and Campbell, 2002b). Briefly, 10 μm frozen sections of cold phosphate buffer saline (PBS) perfused spinal cord tissues mounted on glass slides were fixed for 5 min in acetone/methanol (1:1) and washed thoroughly in PBS before non-specific binding was blocked in antibody diluent (Cell Signaling Technology, Danvers, MA, United States) for 30 min at 4°C. The following primary antibodies were diluted in antibody diluent and incubated overnight at 4°C: rat monoclonals reactive to CD31 (MEC13.3; 1:500), α5 integrin (5H10-27 (MFR5); 1:100), CD45 (1:300), MECA-32 (1:100), and Mac-1 (M1/70; 1:100), all from BD Pharmingen (La Jolla, CA, United States); mouse monoclonal anti-GFAP-Cy3 conjugate (1:2000; Sigma-Aldrich, St. Louis, MO, United States), hamster monoclonal reactive for CD31 (2H8; 1:500) from Abcam (Cambridge, MA, United States), rabbit polyclonals reactive to fibronectin (1:1000; Sigma-Aldrich), and ZO-1 (1:1500) and fibrinogen (1:2000) (both from Millipore, Temecula, CA, United States). Fluoromyelin-red (1:50) was obtained from Invitrogen (Carlsbad, CA, United States). After several rounds of washing with PBS, tissues were further incubated with the following secondary antibodies diluted in antibody diluent and incubated for 2 h at 4°C: Cy3-conjugated anti-rat and anti-rabbit from Jackson Immunoresearch (West Grove, PA), and Alexa Fluor 488-conjugated anti-rat and anti-hamster from Invitrogen (all secondaries at 1:500). Slides were then washed and mounted in mounting medium containing DAPI (Sigma-Aldrich). Labeling with rat IgG control antibody demonstrated lack of vessel autofluorescence (see Supplementary Material).

### Image Analysis

Images were acquired using a 2X, 10X, or 20X objective on a Keyence 710 fluorescent microscope. All analysis was performed in the lumbar spinal cord. For each antigen, images of at least three randomly selected areas were taken at 10X or 20X magnification per tissue section and three sections per spinal cord analyzed to calculate the mean for each animal (*n* = 4 mice per group). For each antigen in each experiment, exposure time was set to convey the maximum amount of information without saturating the image and was maintained constant for each antigen across the different experimental groups. Vascular integrity was evaluated by measuring extravascular leakage of fibrinogen, as measured by the total area of fibrinogen staining per field of view (FOV). Total vascular area (CD31), vascular expression of α5 integrin and fibronectin and leukocyte infiltration as indicated by levels of CD45 and Mac-1 and extent of myelination by fluoromyelin was evaluated by measuring the total area of fluorescence for each marker per FOV. Vascular density was evaluated by counting all the vessels per FOV. The number of MECA-32-positive vessels per FOV was counted in four randomly selected areas in images captured at 10X or 20X magnification per tissue section and three sections per spinal cord analyzed to calculate the mean for each animal (*n* = 4 mice per group). The percentage of vessels expressing ZO-1 was quantified in a similar manner by counting the number of ZO-1 + vessels/total number of vessels. All data analysis was performed using NIH Image J software. This analysis was performed using four animals per condition per experiment, and the results expressed as the mean ± SEM.

### Cell Culture

Pure cultures of mouse microglia were obtained by mechanical shaking of mixed glial cultures (MGC) as described previously (Milner and Campbell, 2002a). Briefly, forebrains from post-natal mice (days 0–2) were stripped of meninges, chopped into small chunks and dissociated in papain before cultured for ∼10 days on ploy-D-lysine (Sigma-Aldrich)-coated T75 tissue culture flasks (Falcon, Franklin, NJ) in DMEM (Sigma) supplemented with 10% fetal bovine serum (Sigma). After establishment of the astrocyte monolayer (7–10 days), the flasks were shaken for 1 h to obtain the loosely adherent microglia. Microglia were counted by hemocytometer and plated at a density of 5 × 10^4^ cells/well in uncoated 24-well plates (Nunc, Naperville, IL, United States) and maintained in the same medium the MGC were cultured in. The purity of these microglial cultures was >99% as determined by Mac-1 positivity in fluorescent immunocytochemistry. Microglia were cultured overnight, then switched to serum-free N2 medium (DMEM supplemented with N2 (Sigma) and grown in the presence or absence of 10 ng/ml TNF-α (R&D) and 15 nM recombinant murine 3K3A-APC that was produced and purified as previously described (Fernandez et al., 2005). After 4 h incubation, 4–6 phase pictures were taken of each condition using a Zeiss Axio Observer microscope. As previously described (Milner and Campbell, 2002a), under basal N2 conditions, microglia displayed two types of morphology: either round (amoeboid) or elongated spindle-shaped cells with one long process extended at both ends. In contrast, cultures treated with APC contained a much higher % of cells displaying a more complex arborized form (ramified). To quantify this marked switch in morphology, we counted the number of cells per FOV that displayed more than 4 process extensions and presented or data as the% of cells with a ramified morphology. Counts were *manually performed* with 4 FOVs analyzed per condition within each experiment and 4 separate experiments performed. The percentage of ramified microglia was quantified and data expressed as the mean ± SD.

### Gel Zymography

Gelatin zymography to detect MMP-9 activity was performed as previously described (Heo et al., 1999). Microglial cells were plated at a density of 5 × 10^4^ cells/well in uncoated 24-well plates and cultured in the presence or absence of 10 ng/ml TNF-α (R&D) and different concentrations of recombinant murine 3K3A-APC (0, 3, 15, and 75 nM). After 2 days culture, microglial supernatants were collected and analyzed for gelatinolytic activity. Positive controls for MMP-9 (obtained from R&D) were included. For quantification, gels were scanned using a Bio-Rad VersaDoc imaging system (Hercules, CA, United States) and band intensities quantified using NIH Image J software. Each experiment was repeated a minimum number of four times and the data expressed as mean ± SD.

### Statistical Analysis

To measure the impact of APC on clinical score, data were analyzed using one-way analysis of variance (ANOVA) followed by *post hoc* Student’s “*t*”-test, in which *p* < 0.05 was defined as statistically significant (*n* = 19 mice per group). In histological studies, all analyses were performed with NIH Image J software, using four animals per condition per experiment, and the results expressed as the mean ± SEM. Statistical significance of histological data was assessed using one-way analysis of variance (ANOVA) followed by Tukey’s multiple comparison *post hoc* test, in which *p* < 0.05 was defined as statistically significant. For analysis of the impact of APC on microglial morphological phenotype or MMP-9 expression *in vitro*, experiments were repeated a minimum number of four times and the data expressed as the mean ± SD. Statistical significance was assessed using ANOVA followed by the Student’s paired “*t*”-test, in which *p* < 0.05 was defined as statistically significant.

## Results

### APC Suppressed the Progression of EAE Both at the Clinical and Histopathological Levels

**FIGURE 1:**
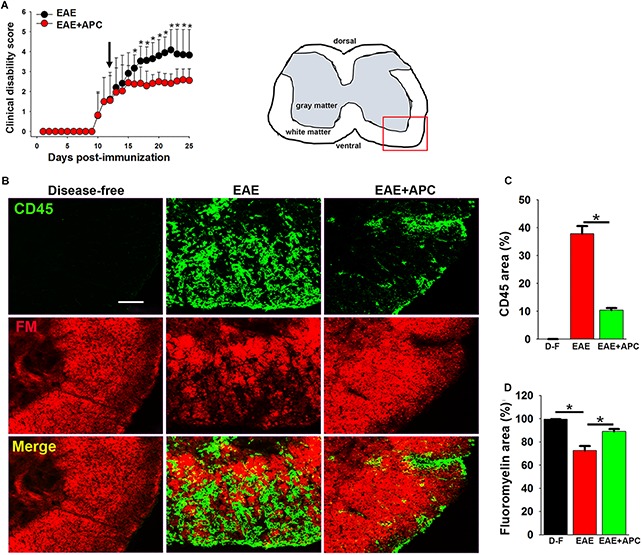
APC suppressed the progression of EAE, both clinically and histopathologically. **(A)** The impact of APC on EAE clinical severity. Once mice acquired a clinical score of 2 they received daily injections of vehicle (EAE) or APC (EAE + APC) as indicated by the arrow, and clinical scores subsequently evaluated at daily intervals. All points represent the mean ± SD (*n* = 19 mice per group in 3 separate experiments). Note that compared to vehicle controls, after 4 days treatment, APC suppressed the progression of clinical score at all time-points for the duration of the experiment. *p < 0.05. **(B)** Frozen sections of lumbar spinal cord taken from disease-free, EAE-vehicle control or EAE + APC mice at the peak phase of EAE were stained for the inflammatory leukocyte marker CD45 (AlexaFluor-488) and fluoromyelin-red (FM). Images were captured in the ventral region as depicted by the red box in the schematic image. Scale bar = 100 μm. **(C,D)** Quantification of CD45 **(C)** and fluoromyelin **(D)** fluorescent signal under disease-free (D–F), or at peak phase of EAE in mice receiving vehicle (EAE) or APC (EAE + APC). Results are expressed as the mean ± SEM (*n* = 4 mice/group). Note that APC markedly suppressed CD45 + leukocyte infiltration and protected against demyelination. *p < 0.05.

The protective potential of APC was evaluated in the chronic EAE model of MS, in which C57BL/6 mice were immunized with the MOG_35__–__55_ peptide as previously described (Crocker et al., 2006; Milner et al., 2007). As shown in Figure 1A, mice began developing signs of clinical disease between 12 and 14 days post-immunization. When each mouse reached a clinical score of 2 (equivalent to hindlimb weakness), they were randomly allocated to one of two different treatment groups: to receive daily intraperitoneal injections of recombinant murine 3K3A-APC (0.2 mg/kg) or PBS vehicle. Rather than use native wild-type APC, in this study we opted to use the 3K3A-APC variant (for simplicity referred to as APC from hereon) that has three point mutations switching lysine for alanine in the protease domain, because this variant retains cytoprotective properties but has lost the majority of its antithrombotic activity, thus minimizing the potential deleterious side-effect of bleeding (Mosnier et al., 2004, 2007). As shown in Figure 1A, after four days of treatment, APC markedly suppressed the clinical progression of EAE at all time-points for the duration of treatment (2 weeks). As the most severe disease in this EAE model occurs in the dorsal end of the spinal cord, we next examined sections of lumbar spinal cord for evidence of neuroinflammation. Histopathological assessment of lumbar spinal cord tissue at the peak of EAE disease (between 21 and 25 days post-immunization) with the pan-leukocyte marker CD45 and the myelin marker fluoromyelin (CD45/fluoromyelin dual-IF) revealed that compared to vehicle-treated controls, APC-treated mice showed marked reduction in the extent of CD45 + leukocyte infiltration into the spinal cord (10.42 ± 0.73 vs. 37.82 ± 2.71% total CD45 + area/FOV under vehicle control conditions, *p* < 0.01) and this was associated with preservation of myelin (89.15 ± 2.09 vs. 72.71 ± 3.81% of fluoromyelin area/FOV under vehicle control conditions, *p* < 0.01) (Figures 1B–D). This demonstrates that APC suppressed EAE progression, both at the clinical and histopathological levels.

### APC Suppresses Vascular Breakdown and Induction of Endothelial MECA-32 Expression

**FIGURE 2:**
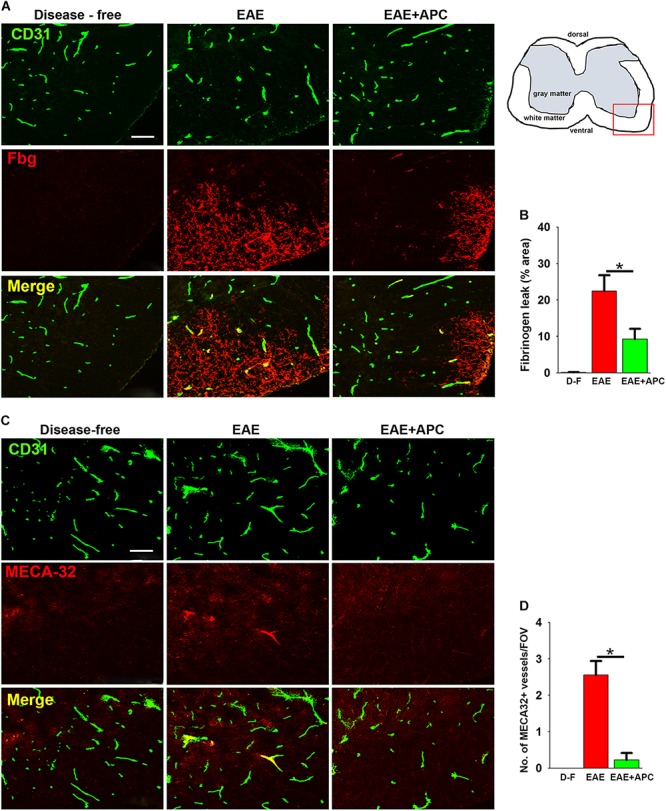
APC protects against loss of vascular integrity during EAE progression. **(A,C)** Frozen sections of lumbar spinal cord taken from disease-free, EAE-vehicle control or EAE + APC mice at the peak phase of EAE were stained for CD31 (AlexaFluor-488) and fibrinogen (Fbg) (Cy-3) in panel A or CD31 (AlexaFluor-488) and MECA-32 (Cy-3) in **(C)**. Images were captured in the ventral region as depicted by the red box in the schematic image. Scale bar = 100 μm. **(B,D)** Quantification of fibrinogen leakage **(B)** and MECA-32 expression **(D)** under disease-free (D–F), or at peak phase of EAE in mice receiving vehicle (EAE) or APC (EAE + APC). Results are expressed as the mean ± SEM (*n* = 4 mice/group). Note that APC markedly suppressed fibrinogen leakage as well as endothelial expression of MECA-32. *p < 0.05.

Previous studies have shown that APC promotes vascular integrity in animal models of ischemic stroke (Mosnier et al., 2007; Guo et al., 2009). To examine how APC influences vascular integrity in this EAE model, we performed dual-IF using CD31 as an endothelial cell marker CD31 and extravascular fibrinogen leak as a marker of vascular breakdown (Figures 2A,B). This revealed that while spinal cords of control mice (EAE without APC treatment) had extensive fibrinogen leakage, most notably in spinal cord white matter, mice treated with APC showed reduced levels of fibrinogen leak in their spinal cords (Figure 2A). Quantification revealed that fibrinogen leakage was significantly reduced in mice receiving APC compared to vehicle controls (9.26 ± 2.78 compared to 22.43 ± 4.36% total fibrinogen area/FOV under normoxic conditions, *p* < 0.01) (Figure 2B). Next, in an alternative approach to examine if blood vessels in APC-treated mice have improved barrier properties we examined vascular expression of MECA-32, a protein that is expressed at high levels on endothelial cells in the developing CNS, but then disappears as CNS endothelium matures (Hallman et al., 1995). Interestingly, MECA-32 is re-expressed in adult CNS blood vessels during inflammatory, hypoxic and ischemic conditions (Engelhardt et al., 1994; Sparks et al., 2000; Li et al., 2012), implying that MECA-32 re-expression on CNS blood vessels occurs when vascular integrity is compromised. Our staining revealed that while no MECA-32 staining was detected in disease-free spinal cord, MECA-32 expression was induced on a sub-population of spinal cord blood vessels in vehicle-treated EAE controls (Figures 2C,D), but of note, significantly less MECA-32 expression was detected on blood vessels in mice treated with APC (0.22 ± 0.19 compared to 2.55 ± 0.38 MECA-32 + vessels/FOV, *p* < 0.01). Taken together, these two lines of data demonstrate that spinal cord blood vessels in APC-treated mice show less vascular breakdown.

### APC Attenuates Loss of the Endothelial Tight Junction Protein ZO-1 During EAE

**FIGURE 3:**
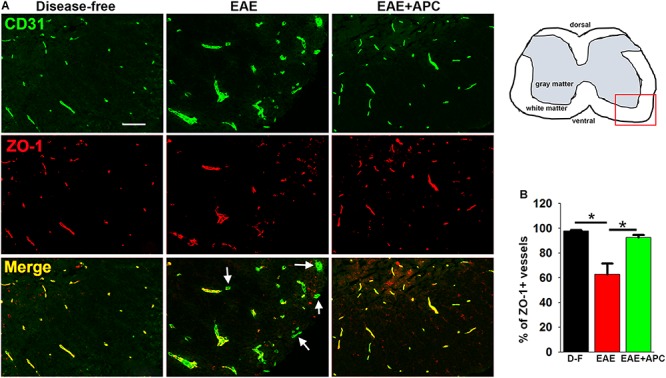
APC protects against loss of the endothelial tight junction protein ZO-1 during EAE. **(A)** Frozen sections of lumbar spinal cord taken from disease-free, EAE-vehicle control or EAE + APC mice at the peak phase of EAE were stained for CD31 (AlexaFluor-488) and ZO-1 (Cy-3). Images were captured in the ventral region as depicted by the red box in the schematic image. Scale bar = 100 μm. **(B)** Quantification of the % of vessels expressing ZO-1. Results are expressed as the mean ± SEM (*n* = 4 mice/group). Note that under disease-free (D–F) conditions, all blood vessels co-expressed ZO-1 as confirmed by the yellow color in the merged image. In contrast, at the peak of EAE disease, many of the blood vessels in the vehicle control group lacked ZO-1 expression (marked by arrows) and illustrated by the green color in the merge image. In contrast to vehicle control mice, APC protected against loss of ZO-1. *p < 0.05.

Vascular integrity of CNS blood vessels is highly dependent on endothelial expression of tight junction proteins such as ZO-1 which serve to form tight connections between adjacent endothelial cells (Huber et al., 2001; Wolburg and Lippoldt, 2002; Ballabh et al., 2004). The importance of these proteins is illustrated by the finding that endothelial expression of tight junction proteins at cell-cell borders is disrupted both in MS and in EAE (Kirk et al., 2003; Bennett et al., 2010; Errede et al., 2012). To examine how APC influences endothelial expression of ZO-1 in EAE, we performed dual-IF of CD31/ZO-1 on spinal cord sections taken from mice at the peak stage of EAE that had been treated with APC or vehicle controls. As expected, under disease-free conditions, ZO-1 co-localized tightly with the endothelial cell marker CD31 on all spinal cord blood vessels (Figure 3A). However, during the peak stage of EAE, a significant number of blood vessels showed total or partial loss of ZO-1 expression (reduced to 62.69 ± 8.89% of ZO-1 + vessels compared to 97.75 ± 2.45% of ZO-1 + vessels under disease-free conditions, *p* < 0.01). In contrast, blood vessels in APC-treated mice largely maintained expression of ZO-1 such that the proportion of vessels expressing ZO-1 in APC-treated mice was significantly higher than vehicle controls (92.57 ± 2.09% vs. 62.69 ± 8.89%, *p* < 0.01). This demonstrates that APC prevented loss of the endothelial tight junction protein ZO-1 during EAE pathogenesis.

### APC Treatment Has No Discernible Influence on Angiogenic Remodeling in EAE

**FIGURE 4:**
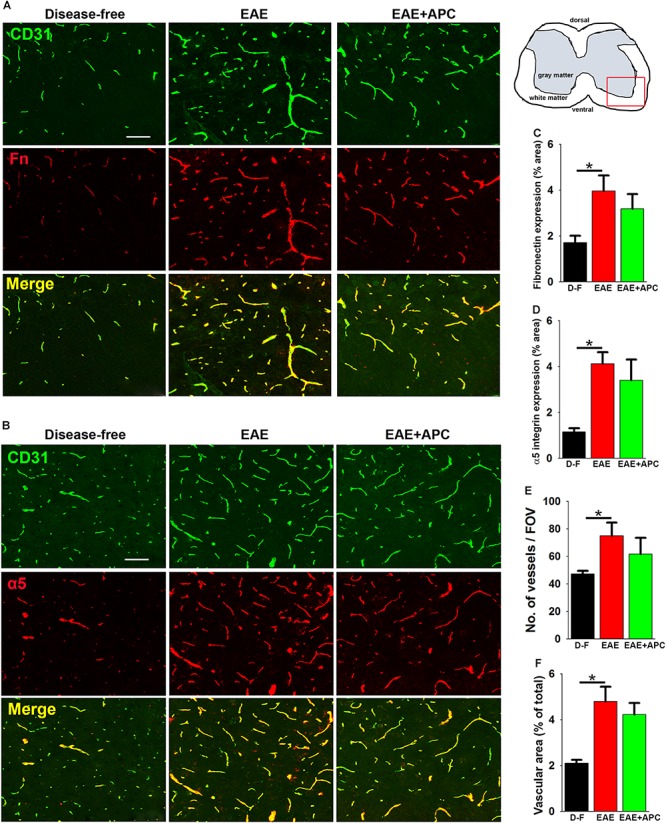
The impact of APC on vascular remodeling during EAE progression. **(A,B)** Frozen sections of lumbar spinal cord taken from disease-free, EAE-vehicle control or EAE + APC mice at the peak phase of EAE were stained for CD31 (AlexaFluor-488) and fibronectin (Fn) (Cy-3) in panel A or CD31 (AlexaFluor-488) and α5 integrin (Cy-3) in **(B)**. Images were captured in the ventral region as depicted by the red box in the schematic image. Scale bar = 100 μm. **(C,D)** Quantification of fibronectin **(C)** and α5 integrin expression **(D)**. **(E,F)** Quantification of vessel density **(E)** and vascular area **(F)**. All results are expressed as the mean ± SEM (*n* = 4 mice/group). Note that compared to disease-free conditions, blood vessels in the EAE-vehicle control group showed increased expression of fibronectin and α5 integrin, which was associated with enhanced vascular density and area, but of note, APC had no significant impact on these changes. *p < 0.05.

In recent studies we found that a marked angiogenic remodeling response occurs during the pre-symptomatic phase of EAE and that this is associated with upregulated expression of fibronectin and its receptor α5β1 integrin on spinal cord blood vessels (Boroujerdi et al., 2013). In a different model, we showed that chronic mild hypoxia stimulates angiogenic remodeling and interestingly found that APC appears to plays a key role in mediating this response, as functional blockade of APC prevented both upregulation of the fibronectin-α5β1 integrin axis and the associated angiogenic response, while in contrast, exogenous APC enhanced this response (Burnier et al., 2016). In light of these findings, we wondered if the protective effect of APC in EAE might be due to enhanced fibronectin-α5β1 integrin signaling driving a stronger angiogenic response. To examine this concept, we performed dual-IF of CD31/fibronectin and CD31/α5 integrin on spinal cord sections taken from mice at the peak stage of EAE that had received APC or vehicle controls. Consistent with previous reports (Boroujerdi et al., 2013), this showed that EAE was strongly associated with upregulation of both fibronectin (3.97 ± 0.67 vs. 1.71 ± 0.29% fibronectin area/FOV under disease-free conditions, *p* < 0.05), and α5 integrin expression (4.12 ± 0.50 vs. 1.15 ± 0.15% α5 integrin area/FOV under disease-free conditions, *p* < 0.05) (Figures 4A–D). Surprisingly however, compared with vehicle control mice, APC showed no discernible increase in either fibronectin expression (3.19 ± 0.64 vs. 3.97 ± 0.67% fibronectin area/FOV under vehicle conditions), or α5 integrin expression (3.40 ± 0.90 vs. 4.12 ± 0.50% α5 integrin area/FOV under vehicle conditions). To examine whether APC could be enhancing angiogenic remodeling via another mechanism, we next examined whether APC influenced vascular density and total vascular area at the peak stage of EAE disease. This showed that while spinal cords of mice with peak EAE disease displayed higher vessel density (75.00 ± 9.60 vs. 47.11 ± 5.11 vessels/FOV under disease-free conditions) and higher total vascular area (4.79 ± 0.65 vs. 2.09 ± 0.15% vascular area/FOV under disease-free conditions) compared to disease-free controls, surprisingly, compared with vehicle controls, APC showed no discernible increase in either vessel density (61.67 ± 11.89 vs. 75.00 ± 9.60 vessels/FOV under vehicle conditions) or total vascular area (4.22 ± 0.51 vs. 4.79 ± 0.65% vascular area/FOV under vehicle conditions). Taken together, these data argue against the notion that APC protects against EAE progression by enhancing angiogenic remodeling.

### APC Suppresses Microglial Activation in EAE

**FIGURE 5:**
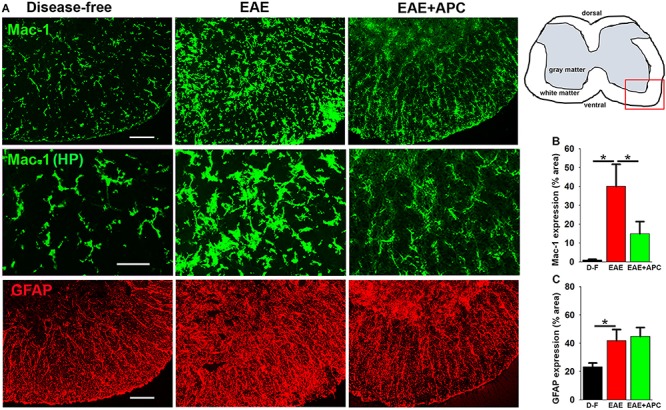
APC suppresses microglial activation during EAE. **(A)** Frozen sections of lumbar spinal cord taken from disease-free, EAE-vehicle control or EAE + APC mice at the peak phase of EAE were stained for Mac-1 (AlexaFluor-488) and GFAP (Cy-3). Images were captured in the ventral region as depicted by the red box in the schematic image. Scale bar = 100 μm except for the high-power images in the middle row = 50 μm. **(B,C)** Quantification of Mac1 **(B)** and GFAP **(C)** expression. Results are expressed as the mean ± SEM (*n* = 4 mice/group). In the higher magnification images (middle row), note that microglia in vehicle-treated EAE mice displayed large cell bodies and much higher Mac-1 expression, typical of highly activated microglia while in contrast, microglia in APC-treated mice showed highly ramified morphology with small cell bodies and low Mac-1 expression, typical of less activated microglia and more similar to those present in disease-free mice. In addition, GFAP levels in EAE mice were strongly enhanced over disease-free conditions but APC had no discernible effect on this expression. *p < 0.05.

Because microglia, the primary immune effector cells resident in the CNS, are thought to play a pivotal role both in the initiation and maintenance of chronic inflammation in MS (Ransohoff, 1999; Trapp et al., 1999), we next examined the influence of APC on microglial activation. As Mac-1 expression levels correlate with degree of microglial activation, we first examined this *in vivo* using Mac-1/GFAP dual-IF. As shown in Figure 5, spinal cords of EAE mice showed strongly elevated levels of microglial Mac-1 expression (40.05 ± 11.74 vs. 0.95 ± 0.36% Mac-1 area/FOV under disease-free conditions, *p* < 0.05), but interestingly, Mac-1 levels in APC-treated mice were markedly lower than vehicle-treated controls (14.90 ± 6.48 vs. 40.05 ± 11.74% Mac-1 area/FOV under vehicle-EAE conditions, *p* < 0.05). Strikingly, compared to disease-free conditions, microglia in vehicle-treated EAE mice displayed large cell bodies and much higher Mac-1 expression, typical of highly activated microglia (see high magnification images in the middle row of Figure 5A). In contrast, microglia in APC-treated mice showed highly ramified morphology with small cell bodies and lower levels of Mac-1 expression, typical of less activated microglia and similar to those present in disease-free mice. Examination of astrocyte activation by GFAP IF revealed that GFAP levels in the spinal cords of mice with established EAE were enhanced over disease-free conditions (41.82 ± 8.07 vs. 23.51 ± 2.81% Mac-1 area/FOV under disease-free conditions, *p* < 0.05), but treatment with APC had no significant effect on the overall level of GFAP expression (Figure 5C).

### APC Also Suppresses Microglial Activation and MMP-9 Expression *in vitro*

**FIGURE 6:**
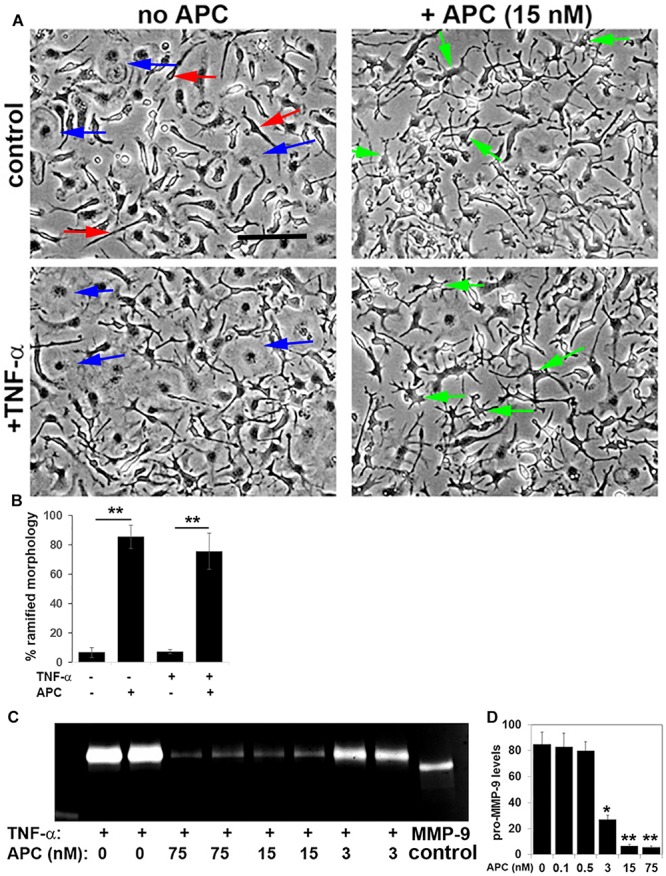
APC suppresses microglial activation and MMP-9 expression *in vitro*. **(A)** Pure cultures of microglia were cultured in the presence or absence of 10 ng/ml TNF-α and/or 15 nM APC. Under control conditions, microglia occupied a mix of amoeboid (blue arrows) and elongated spindle-shaped cells (red arrows). Note that TNF-α promoted the amoeboid phenotype, typical of activated cells, but APC promoted a morphological transformation of microglia into ramified highly arborized cells (green arrows), typical of quiescent microglia. **(B)** Quantification of the percentage of microglia with a ramified morphology under different conditions. Results are expressed as the mean ± SEM (*n* = 4 images per condition and 4 experiments). **p < 0.01. **(C)** Gel zymography of cell culture supernatants taken from microglia that had been treated with TNF-α in the presence of different doses of APC. **(D)** Quantification of MMP-9 levels. Results are expressed as the mean ± SEM (*n* = 2 samples per condition and 4 experiments). Note that at doses of 75 or 15 nM, APC almost completely blocked TNF-α induction of microglial MMP-9 expression. *p < 0.05, **p < 0.01.

While our *in vivo* data show that APC suppresses neuropathology and microglial activation in the EAE model, it is difficult to know whether the effect we observe on microglial activation is a direct or secondary response. Therefore, in order to examine if APC directly influences microglial activation, we studied this process in pure cultures of primary mouse microglia, which were derived by mechanical separation from MGC set up from post-natal mice brains, as previously described (Milner and Campbell, 2002a). Following separation from MGC, microglia were cultured overnight, then switched to serum-free N2 medium and stimulated with APC. After 4 h stimulation with 15 nM APC, clear morphological changes in microglia became evident (Figure 6A), with cells switching from a predominantly flattened morphology under control conditions to ramified cells having multiple fine cytoplasmic extensions in the presence of APC. In addition, while the pro-inflammatory cytokine TNF-α enhanced cell flattening and the appearance of an amoeboid morphology typical of the activated phenotype, APC prevented this transformation and maintained the majority of microglia in the ramified morphology, typical of quiescent microglia. As MMP-9 levels are strongly upregulated upon microglial activation (Kauppinen and Swanson, 2005; Milner et al., 2007; Crocker et al., 2008) and are known to play important pathogenic roles in degrading vascular basement membrane extracellular matrix proteins as well as myelin proteins of oligodendrocytes, we next examined how APC regulates microglial MMP-9 expression. Pure microglia were stimulated with TNF-α to promote microglial activation, then co-incubated with different doses of APC. Two days later, MMP-9 levels within supernatants were analyzed by gel zymography (Figures 6C,D). This showed that consistent with previous findings, TNF-α strongly stimulated microglial MMP-9 expression (Crocker et al., 2008). Interestingly, at doses of 75 or 15 nM, APC almost completely blocked TNF-α induction of microglial MMP-9. This inhibition was still evident at the reduced dose of 3 nM APC but diminished at lower doses. Taken together, these *in vitro* studies demonstrate that a dose of 15 nM, APC effectively blocks microglial activation as assessed both at the morphological level and expression of MMP-9. These findings demonstrate that APC directly suppresses microglial activation, though it does not exclude the possibility that some of the anti-inflammatory actions of APC seen *in vivo* may also be secondary, such as prevention of BBB disruption.

## Discussion

Inspired by the beneficial effect of APC in stroke models (Thiyagarajan et al., 2008; Wang et al., 2013a, b), recent studies have investigated APC’s therapeutic potential in the mouse model of MS, experimental autoimmune encephalomyelitis (EAE), though outcomes have been mixed. Interestingly, while enhancement of APC levels either directly by administration of exogenous APC or via stimulation of the thrombomodulin pathway, conferred protection against EAE (Han et al., 2008; Verbout et al., 2015; Wolter et al., 2016), surprisingly, antibody neutralization of endogenous APC also achieved the same result (Alabanza et al., 2013). Based on this apparent contradiction, the goals of the current study were to clarify the therapeutic potential of APC in the chronic progressive form of EAE, and to determine mechanistically how APC mediates its protective effects. The main findings from our studies were as follows: (1) APC suppressed the progression of EAE, both at the clinical and pathological level, resulting in reduced levels of leukocyte infiltration and concomitant protection against loss of myelin, (2) while APC had no effect on angiogenic remodeling in the EAE model, it prevented loss of vascular integrity which was concomitant with maintained endothelial expression of the tight junction protein ZO-1, and (3) APC suppressed microglial activation both *in vivo* and *in vitro*. These findings build on the work of others in demonstrating strong therapeutic potential for APC in the treatment of inflammatory demyelinating disease and suggest that enhancement of vascular integrity and suppression of microglial activation may be important mediators of this protection.

### The Impact of APC on Vascular Protection

One of the major findings of our study was that APC significantly reduced the extent of vascular breakdown in the EAE model, as shown by reduced degree of fibrinogen leak into the parenchyma and suppression of MECA-32 induction on endothelial cells. These findings support the work of others who have demonstrated that APC is vasculoprotective in animal models of ischemic stroke and sepsis (Mosnier and Griffin, 2007; Mosnier et al., 2007; Schuepbach et al., 2009), as well as in cell culture models of human endothelial cell monolayers (Feistritzer and Riewald, 2005; Finigan et al., 2005). Recent work has shed light on the signaling mechanisms underpinning APC’s vasculoprotective influence by revealing that APC promoted vascular integrity of an endothelial monolayer *in vitro* and that infusion of APC enhanced vascular barrier integrity in a sepsis model in wild-type mice but not in transgenic mice deficient in protease activated receptor-1 (PAR-1) mice (Schuepbach et al., 2009). In future studies we plan to examine whether APC’s protective influence on EAE disease progression is lost in PAR-1 KO mice.

Based on our previous findings that during the pre-symptomatic phase of EAE, a marked vascular remodeling response occurs (Boroujerdi et al., 2013), and that APC plays a key role in mediating cerebrovascular remodeling in response to chronic mild hypoxia (Burnier et al., 2016), we wondered if the protective effect of APC in EAE might, in part, be the result of upregulated fibronectin-α5β1 integrin signaling driving an enhanced angiogenic response. However surprisingly, APC-treated mice showed no appreciable differences in their vascular expression of fibronectin or α5β1 integrin or their overall level of vascularity, compared to vehicle-treated control mice. One possibility is that because such a strong vascular remodeling response occurs during the development of EAE (Boroujerdi et al., 2013), perhaps vascular remodeling is running at near-maximal level, precluding further enhancement of this process.

### The Impact of APC on Microglial Activation

Another important finding of our study was that APC suppressed microglial activation both *in vivo* and *in vitro*. Once stimulated, microglia are activated into migratory phagocytic cells that orchestrate the influx of infiltrating leukocytes via cytokine and chemokine communication (Hanisch and Kettenmann, 2007). However if persistently activated, microglia induce excessive tissue damage, and the current consensus is that microglia play a critical role both in the initiation and maintenance of MS pathogenesis (Ransohoff, 1999; Trapp et al., 1999). Our observation that APC suppresses microglial activation *in vivo* suggests two possibilities; either APC is directly suppressing microglial activation *per se*, or this is an indirect consequence of APC reducing BBB breakdown, resulting in less influx of microglial-activating factors such as fibrinogen, fibronectin or vitronectin (Adams et al., 2007; Milner et al., 2007), or via activation of other CNS cell types, such as astrocytes. Interestingly, while the increase in total GFAP levels induced by EAE disease were not affected by APC, we did observe some localized differences in GFAP expression and organization in APC treated mice, suggesting that the impact on APC on glial cells in the CNS might not be entirely microglial-selective. While it is hard to determine whether APCs effects on microglial suppression *in vivo* are primary or secondary, our *in vitro* studies demonstrated that APC directly suppressed microglial activation because it promoted morphological transformation of microglia from the classic activated amoeboid morphology into the ramified form, typical of quiescent microglia, and also suppressed expression of the pro-inflammatory protease MMP-9. These data are consistent with previous findings that APC suppressed microglial activation in an animal model of amyotrophic lateral sclerosis (ALS), a protective effect that was shown to correlate with downregulation of superoxide dismutase-1 (SOD-1) in both microglial cells and neurons (Zhong et al., 2009). Taken together, our findings demonstrate that APC directly suppress microglial activation, though it does not exclude the possibility that some of the anti-inflammatory actions of APC seen in the EAE model *in vivo* may also be secondary to other effects, such as prevention of BBB disruption.

## Conclusion

The goals of this study were to evaluate the therapeutic potential of APC in the chronic progressive form of EAE and determine mechanistically how APC mediates protection. We found that APC suppressed the clinical progression of EAE, both at the clinical and pathological level, resulting in reduced levels of leukocyte infiltration and concomitant demyelination. IF analysis revealed that APC reduced vascular breakdown which was associated with maintained endothelial expression of the tight junction protein ZO-1. In addition, APC suppressed microglial activation both *in vivo* and *in vitro*. These findings build on the work of others in demonstrating strong therapeutic potential for APC in the treatment of inflammatory demyelinating disease and suggest that enhancement of vascular integrity and suppression of microglial activation are key mediators of this protection.

## Data Availability Statement

The datasets generated for this study are available on request to the corresponding author.

## Ethics Statement

The studies described have been reviewed and approved by The Scripps Research Institute Institutional Animal Care and Use Committee.

## Author Contributions

RK and SH performed the EAE studies, analyzed clinical EAE progression, and performed the histological analysis. JF and JG provided the APC used in this study. RM established the microglial cell cultures and performed the gel zymography. RM and JG conceived of the study and drafted the manuscript. All authors read and approved the final manuscript.

## Conflict of Interest

The authors declare that the research was conducted in the absence of any commercial or financial relationships that could be construed as a potential conflict of interest.

## Abbreviations

APC: Activated protein C
BBB: Blood-brain barrier
BEC: Brain endothelial cells
CFA: Complete Freund’s adjuvant
CNS: Central nervous system
Dual-IF: Dual-immunofluorescence
EAE: Experimental autoimmune encephalomyelitis
ECM: Extracellular matrix
FOV: Field of view
MOG: Myelin Oligodendrocyte Glycoprotein
MS: Multiple sclerosis
PAR-1: Protease activated receptor
SEM: Standard error of the mean
TNF- α: Tumor necrosis factor-alpha
WT: Wild-type
ZO-1: Zonula occludens-1.
