# Supplementary material for: Activated Protein C Attenuates Experimental Autoimmune Encephalomyelitis Progression by Enhancing Vascular Integrity and Suppressing Microglial Activation
Source: Front Neurosci. 2020 Apr 15;14:333. doi: 10.3389/fnins.2020.00333 (PMC7174764; doi:10.3389/fnins.2020.00333)
Supplement: Supplementary file 1 [file Data_Sheet_1.PDF]

Supplemental Figure.

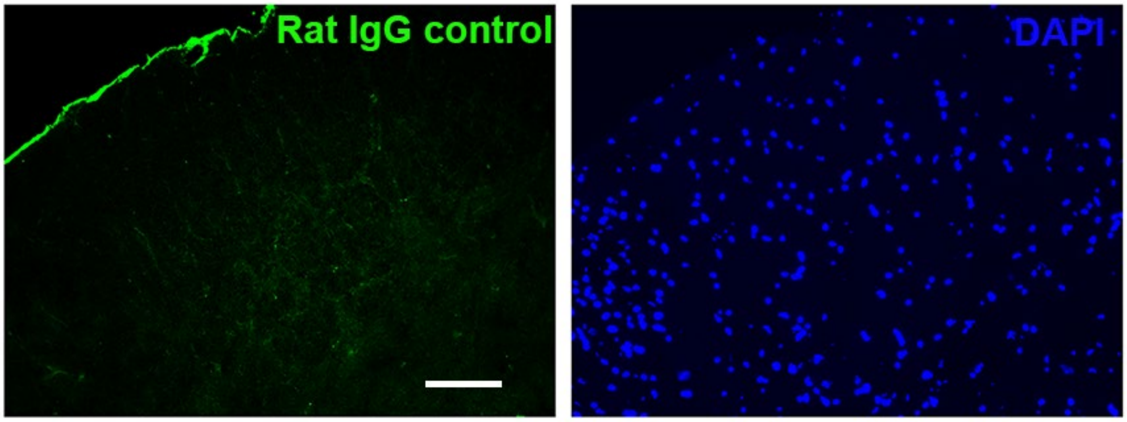

Control IgG immunostaining of the ventral region of lumbar spinal cord. Frozen sections of lumbar spinal cord taken from EAE-vehicle mice at the peak phase of EAE were stained with rat anti-mouse IgG followed by AlexaFluor-488-conjugated anti-rat secondary. DAPI staining of the same field is shown to the right. Scale bar = 100 μm. Note the absence of any vessel autofluorescence.
